# Tripartite motif-containing protein 26 promotes colorectal cancer growth by inactivating p53

**DOI:** 10.21203/rs.3.rs-3782833/v1

**Published:** 2024-01-10

**Authors:** Hua Lu, Zhihui Tan, Hyunmin Ko, Parnia Naji, Rong Zhu, Jieqiong Wang, Shibo Huang, Yi-Wei Zhang, Shelya Zeng

**Affiliations:** Tulane University; Tulane University; Tulane University; Tulane University; Tulane University; Tulane University; Tulane University; Tulane University School of Medicine; Tulane University-school of medicine

**Keywords:** TRIM26, colorectal cancer, p53, ubiquitin ligase, tumor growth, MDM2

## Abstract

Tripartite motif-containing protein 26 (TRIM26) is an E3 ubiquitin ligase that exhibits divergent roles in various cancer types (oncogenic and anti-oncogenic). This study investigates the interaction of TRIM26 with the tumor suppressor protein p53 in colorectal cancer (CRC) cells by performing a comprehensive set of biochemical, cell-based assays, and xenograft experiments. As a result, we found that overexpression of TRIM26 significantly enhances CRC cell proliferation and colony formation, while knockdown of TRIM26 suppresses these processes. Xenograft experiments further validated the tumor-promoting role of TRIM26 in CRC. Supporting this is that TRIM26 is highly expressed in human CRC tissues as revealed by our analysis of the TCGA database. Biochemically, TRIM26 directly bound to the C-terminus of p53 and facilitated its ubiquitination, resulting in proteolytic degradation and attenuated p53 activity independently of MDM2. Also, TRIM26 increased the MDM2-mediated ubiquitination of p53 by binding to MDM2’s C-terminus. This study uncovers the oncogenic potential of TRIM26 in CRC by inhibiting p53 function. Through its ubiquitin ligase activity, TRIM26 destabilizes p53, consequently promoting CRC cell proliferation and tumor growth. These findings shed light on the complex involvement of TRIM26 in cancer and identify this ubiquitin ligase as a potential therapeutic target for future development of CRC treatment.

## Introduction

Tripartite motif-containing protein 26 (TRIM26) is one of the TRIM family members and composed of the N-terminal RING, B-box 2, Coiled coil, and C-terminal PRY-SPRY domains (1). Its gene is located and clustered with six other TRIM family members on chromosome 6p21.33–6p22.2 (2). Although functioning as E3 ubiquitin ligases, TRIMs have been shown to play diverse roles in biological processes, such as cellular proliferation, cell cycle advancement, DNA maintenance, ferroptosis, and autophagy (3, 4). TRIM26’s functions in cancers and their underlying mechanisms just began to be unveiled less than a decade ago. On the one hand, TRIM26 was reported to play a potential tumor suppressive role in some types of human cancers. For example, overexpression of TRIM26 inhibited the growth of non-small cell lung carcinoma cells by suppressing the PI3K/AKT signaling pathway (5) and also, led to suppression of proliferation, metastasis, and growth of papillary thyroid cancer cells (6). Similarly, TRIM26 could also act as a tumor suppressor of hepatocellular carcinoma (HCC), and its downregulation was associated with poor prognosis of HCC (7). This result was later validated by another study showing that TRIM26 can suppress HCC growth and migration by ubiquitinating and degrading Zinc-finger E-box-binding homebox1 (ZEB1) (8), an oncogenic transcriptional factor crucial for HCC growth and metastasis (8–10). On the other hand, more recent studies showed that TRIM26 surprisingly plays an oncogenic role in different types of tumors. Knockdown of TRIM26 led to the inhibition of proliferation, migration, and invasion of bladder cancer cells by impeding the AKT/GSK3b/b-catenin pathway (4). Fascinatingly, TRIM26 was found to stabilize SOX2 protein and enhance its oncogenic activity in glioblastoma via its C-terminal PRYSPRY domain without engaging its Ring domain and E3 ligase activity (11). These studies suggest that TRIM26 appears to play both oncogenic and anti-oncogenic roles, pending on types of tissues, cells, or tumors. However, no study has explored the possibility of whether TRIM26 might regulate the functions of p53 that is the most important genome guardian as a tumor suppressor.

The tumor suppressor p53 can activate the expression of various genes crucial for cell-cycle control, senescence, DNA repair, apoptosis, necrosis, autophagy, and ferroptosis in response to various stressors (12). p53 protein is composed of an N-terminal transactivational domain (TAD), a central DNA binding domain (DBD), a tetramerization domain, and a C-terminal regulatory region (12) and functions as a homotetrameric transcription factor (13, 14). The p53 stability is regulated by MDM2 via ubiquitination-dependent proteolysis in a negative feedback fashion (15–17). In addition to MDM2, p53 can be ubiquitinated by other ubiquitin ligases under different cellular and pathological conditions (18–20). When searching for possible new p53 regulators, we identified TRIM26 as another p53 E3 ligase. Our further studies on the role of TRIM26 in regulation of p53 reveal that this Ring E3 ligase can ubiquitinate p53 and lead to its proteolytic degradation, consequently inactivating p53 and promoting the growth and proliferation of colorectal cancer cells in culture and in xenograft. Additionally, it directly interacts with both p53 and MDM2, thereby further enhancing p53 ubiquitination facilitated by MDM2. TRIM26 is highly expressed in colorectal adenocarcinomas as revealed by our analysis of the TCGA database. Thus, our results as further described below demonstrate that TRIM26 acts as an oncoprotein to promote colorectal cancer growth by inactivating p53.

## Results

### TRIM26 promotes proliferation and growth of colorectal cancer cells

**Figure 1:**
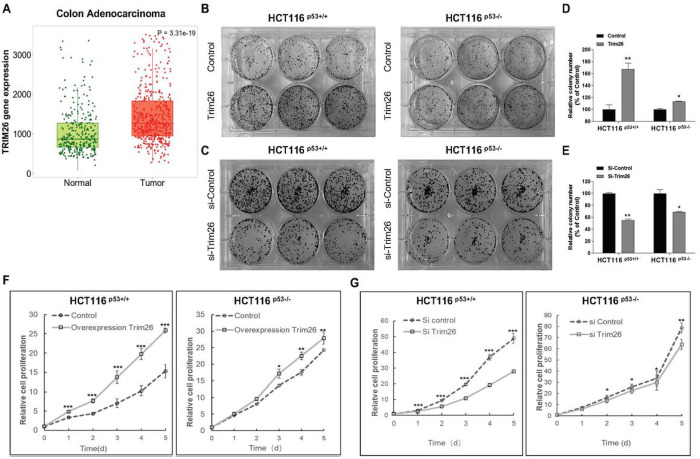
Trim26 is overexpressed in colon adenocarcinoma and promotes cell growth and proliferation. (**A**) Trim26 mRNA expression determined using RNA-sequencing in normal colon tissues (*n* = 315) and colon cancer (*n* = 469) from TNMplot.com. (**B-C**) TRIM26 promotes colony formation. The cells were transfected with Flag-Trim26 or Si-Trim26 in HCT116 (p53^+/+^) and HCT116 (p53^−/−^) and seeded in 6-well plates for 14 days: (**B**): Cell colony formation after overexpression of Trim26. (C): Cell colony formation after knockdown of Trim26. Histograms indicate the relative colony number (**D**: overexpression of Trim26, E: knockdown of Trim26 (*P<0.05, **P < 0.01). (**F-G**) TRIM26 promotes cell proliferation, partially in a p53-dependent manner in colon cancer cell. Cell proliferation after (**F**) overexpression and (**G**) knockdown of Trim26 in HCT116^(p53+/+)^ and HCT116^(p53−/−)^; The cells were transfected with Flag-Trim26 or Si-Trim26 for 24 h and then split into 96-well plates, CCK-8 were added for 1h and OD-450 was measured each day and for 5 days. *P<0.05, **P < 0.01, ***P < 0.001.

Since TRIM26 has been shown to play both oncogenic and anti-oncogenic roles pending on types of cells and cancers (4, 6), we wondered if it might affect the growth of colorectal cancer (CRC) cells once we identified this protein as a potential p53-binding protein as described later in this report. Our TCGA database analysis of the Trim26 gene expression as shown in RNA-sequencing data from TNMplot.com (Fig. 1A) showed that its RNA level is significantly higher in colon adenocarcinoma tissues than that in healthy colon tissues. This result suggested that TRIM26 might be favorable to the development of this cancer. The same pattern was detected in lung cancer and skin melanoma tissues (SFig. 1A). Thus, to test if TRIM26 might play a role in CRC cell survival and proliferation, we performed a set of colony formation and survival assays in HCT116 CRC cells (see Materials and Methods). As shown in Figs. 1B–1E, overexpression of TRIM26 promoted (B, D), but knockdown of TRIM26 suppressed (C, E), colony formation of CRC cells. Notably, this effect was much more pronounced and more statistically significant in p53-proficient HCT116 cells than p53-null HCT116 cells (Figs. 1B and 1C). As part of result validation, p53-containing SK-Mel-5 and SK-Mel-147 cell lines, derived from skin melanoma, underwent testing, yielding outcomes consistent with those obtained from the HCT cell line (SFigs. 2B and 2C). Consistent with these results, overexpression of TRIM26 also enhanced (Fig. 1F), while knockdown of TRIM26 inhibited (Figs. 1G), proliferation of CRC cells. Again, this effect appeared to be in part dependent of p53, as it was more significant in HCT116^p53+/+^ than HCT116^p53−/−^ cells when TRIM26 was knocked down (Figs. 1F and 1G). These results suggest that TRIM26 can promote the proliferation and growth of CRC cells in part dependently of p53, though it might also possess a p53-independent oncogenic activity in the cancer cells.

### TRIM26 promotes growth of xenograft colorectal tumors by suppressing p53 activity

**Figure 2:**
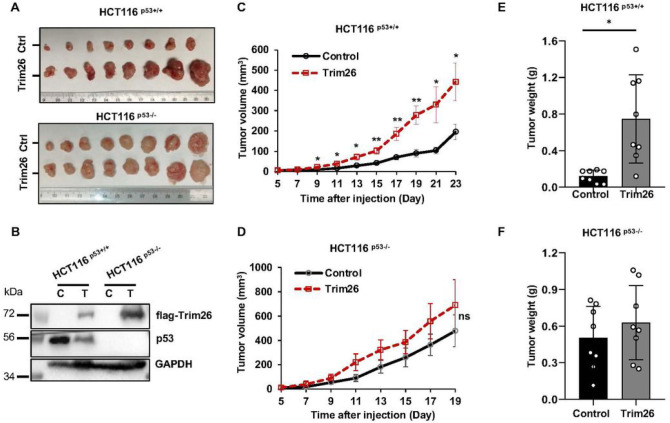
Trim26 overexpression induced tumor progression in xenograft tumor models in a p53 dependent way. Stable cell lines were constructed in colon cancer cells HCT116^(p53+/+)^ and HCT116^(p53−/−)^ using PLVX-flag-Trim26 as positive and PLVX-vector as control. Stable cells were injected to the Nude mice 8 mice each group. The tumor volumes were measured 5 days after the cell injection and tumor weights were measured for 23 days in HCT116^(p53+/+)^ group and for 21 days in HCT116^(p53−/−)^ group. (**A**): photographs of the xenograft tumors retrieved; (**B**): The overexpressed Trim26 protein level was confirmed by WB analysis immediately following implantation of Trim26-overexpressed HCT116 stable cells;(**C, D**): The average tumor volumes at the indicated time are depicted. (**E, F**): tumor weights were measured immediately after isolation. The data are plotted as mean ± SE (n = 8/group); *P < 0.05, **P < 0.01 vs. control.

**Figure 3:**
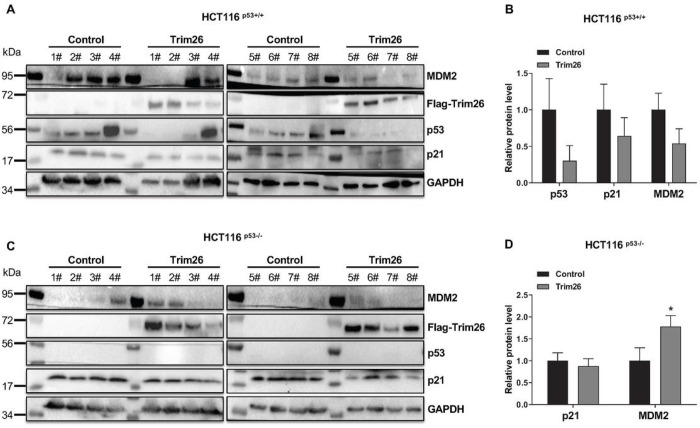
Trim26 overexpression induced tumor progression in xenograft tumor models based on a p53 dependent manner. The protein levels of p53, MDM2, p21 were measured by WB analysis after tumor tissues were isolated. (**A, B**): The protein levels were measured in 8 mice injected stable overexpressed Trim26 or control HCT116^(p53+/+)^ cells, histograms indicate the relative average expression level. (**C, D**): The protein levels were measured in 8 mice injected stable overexpressed Trim26 or control HCT116^(p53−/−)^ cells, histograms indicate the relative average expression level. The data are plotted as mean ± SE (n = 8/group); *P < 0.05 vs. control.

To further validate the potential oncogenic role of TRIM26 as suggested in our cell-based assays above (Fig. 1), we performed a xenograft experiment by inoculating HCT116^p53+/+^ or HCT116^p53−/−^ cells that harbored ectopically expressed TRIM26. The growth of HCT116 cells-derived xenograft tumors was monitored for 3 weeks or so and then harvested for further analyses on day 23 or day 21 as indicated in Fig. 2C. As shown in Fig. 2, overexpression of TRIM26 (Fig. 2B) in the CRC cells markedly promoted the growth of the xenograft tumors. This promotion was more statistically significant in HCT116^p53+/+^ cells-derived tumors than in HCT116^p53−/−^ cells-derived tumors in terms of tumor volumes (compare 2C to 2D) and tumor weights (compare 2E to 2F). This result indicated that TRIM26 might promote tumor growth by suppressing p53 activity. Indeed, this was the case as ectopic TRIM26 led to the reduction of p53 and p21 protein levels in HCT116^p53+/+^ cells-derived tumors (Figs. 3A and 3B), but not in HCT116^p53−/−^ cells-derived tumors (Figs. 3C and 3D). Consistently, p21 and MDM2 protein levels were also markedly reduced in HCT116^p53+/+^ cells-derived tumors (Fig. 3B), but not in HCT116^p53−/−^ cells-derived tumors (Fig. 3D). Taken together, these results indicate that TRIM26 can promote the growth of human CRC cells-derived xenograft tumors likely by suppressing p53 activity.

### TRIM26 reduces p53 level and activity in colorectal cancer cells.

**Figure 4:**
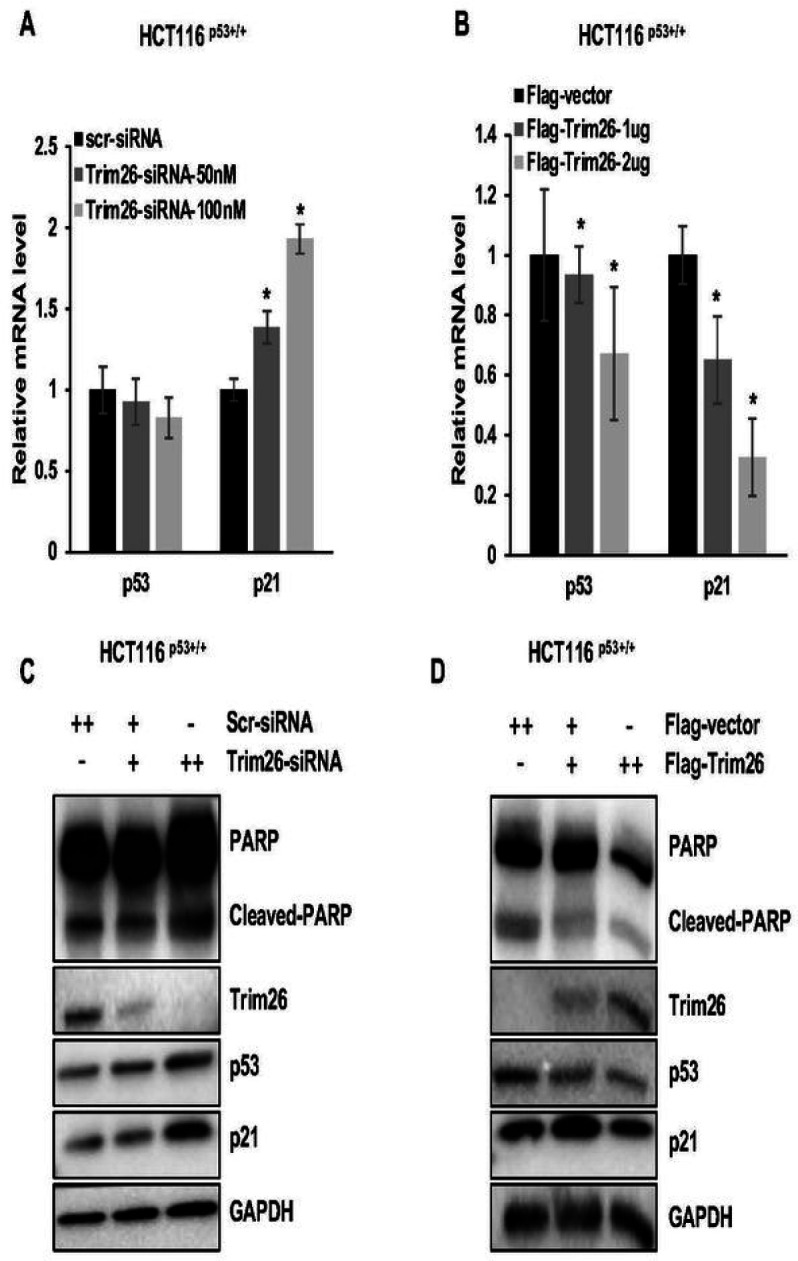
Trim26 inhibits wt p53 in protein level. (**A-B**): p53 and p21 mRNA levels measured by Q-PCR in HCT116^(p53+/+)^ cells. The cells were transfected with Si-Trim26, Si-control, Flag-Trim26 or Flag-control for 48 h and harvested for Q-PCR test with specific primer. (**C-D**): Protein levels after knocking down or overexpression Trim26 in HCT116^(p53+/+)^ cells. The cells were transfected with Si-Trim26, Si-control, Flag-Trim26 or Flag-control for 48 h and harvested for WB analysis with indicated antibodies.

To understand how TRIM26 might regulate p53 level and activity, we conducted a set of cell-based assays by either knocking down or overexpressing TRIM26 in HCT116^p53+/+^ cells. Knockdown of TRIM26 by its specific siRNA induced (Figs. 4A and 4C), but overexpression of TRIM26 reduced (Figs. 4B and 4D), the protein and RNA levels of p21 in a dose-dependent fashion. Interestingly, knockdown of TRIM26 by its specific siRNA induced (Fig. 4C), while overexpression of TRIM26 reduced (Fig. 4D), the protein level of endogenous p53 in these CRC cells. Yet, either approach did not affect the RNA level of p53 significantly (Figs. 4A and 4B). Correspondingly, cleaved PARP was induced by knocking down TRIM26, but reduced by overexpressing TRIM26 in the cells (Figs. 4C, 4D and SFigs. 1A, 1B), suggesting that TRIM26 can suppress p53-dependent apoptosis. Along with these findings, we conducted tests on SK-Mel-5 and SK-Mel-147 cell lines to verify the impact of TRIM26 on p53-dependent apoptosis (SFigs. 3A-C). These results not only were consistent with the xenograft results as shown in Figs. 3A and 3B, but also suggested that TRIM26 might regulate the protein, but not RNA, level of p53.

**Figure 5:**
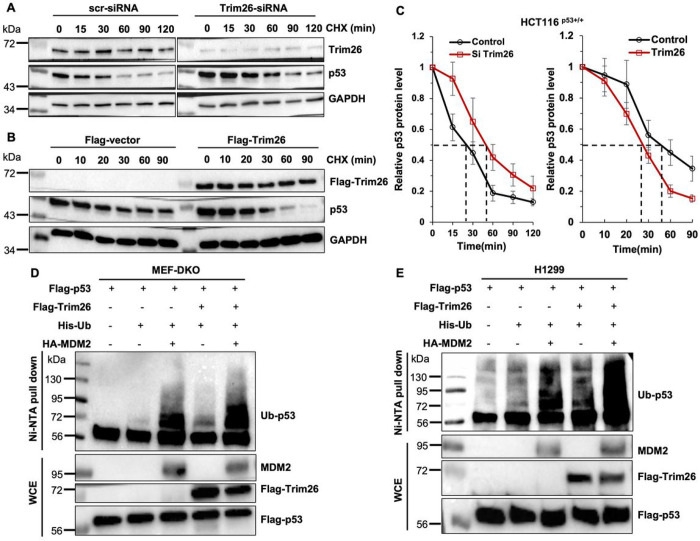
Trim26 increases ubiquitination of wt p53. (**A-B**) Half-life of p53 in Trim26-knockdown and Trim26-overexpressed HCT116^(p53+/+)^ cells. The protein levels of p53 were detected by WB. The HCT116^(p53+/+)^ cells were transfected with Si-Trim26, Si-control, Flag-Trim26 or Flag-control for 48 h, and then treated with 100μg/ml of CHX and harvested at different time points after the treatment for WB analysis with indicate antibodies. Equal amount of proteins (50μg) was loaded in each lane. (**C**) p53-half-lives were quantified by densitometry and plotted against time. (**D-E**) Ubiquitination assays of exogenous wt p53 was conducted in p53- and MDM2-null (**D**) MEF cells and (**E**) H1299 cells with or without Trim26 overexpression. Cells were transfected with plasmids indicated in the figure for 48 h and treated with 20uM MG132 for 6 h before being collected. Proteins were extracted for Ni-NTA beads pulldown assays followed by WB analysis with indicated antibodies. Ubiquitinated proteins and total proteins were detected by WB analysis with indicated antibodies. Equal amount of proteins (50μg) was loaded in each lane.

To test if TRIM26 might affect p53 stability, we assessed the half-life of the p53 protein by either knocking down or overexpressing TRIM26 in HCT116 cells. As shown in Figs. 5A–5C, knockdown of TRIM26 extended the half-life of p53 from ~ 30 minutes to ~ 60 minutes, whereas overexpression of TRIM26 shortened p53’s half-life from ~ 30 minutes to ~ 20 minutes. Of note, because there is a relatively high level of endogenous TRIM26 in HCT116 cells (Fig. 5A), the effect of ectopic TRIM26 on p53’s half-life was not as more pronounced, though still significant, as the effect of depletion of TRIM26 on p53’s half-life (Fig. 5C). These results indicate that TRIM26 can destabilize p53 protein, consequently inhibiting its activity.

### TRIM26 ubiquitinates p53 and also enhances MDM2 ubiquitination of p53.

Since TRIM26 possesses an intrinsic E3 ubiquitin ligase activity (5), next, we tested if TRIM26 might mediate p53 degradation via ubiquitination by conducting a cell-based p53 ubiquitination assay with MDM2 as a positive control in p53/MDM2 double knockout murine embryonic fibroblast (MEF) cells. As shown in Fig. 5D, overexpression of TRIM led to basal level of p53 ubiquitination independently of MDM2. Although its ubiquitination activity toward p53 was not as strong as MDM2’s activity, TRIM26 could further enhance p53 ubiquitination in the presence of MDM2 (last lane of Fig. 5D). These results were repeated in human lung cancer H1299 cells that are p53-deficient (Fig. 5E). Taken together, these results indicate that TRIM26 can mediate p53 proteolytic degradation by ubiquitinating it and boost MDM2-mediated p53 ubiquitination.

### TRIM26 binds to C-termini of p53 and MDM2

**Figure 6:**
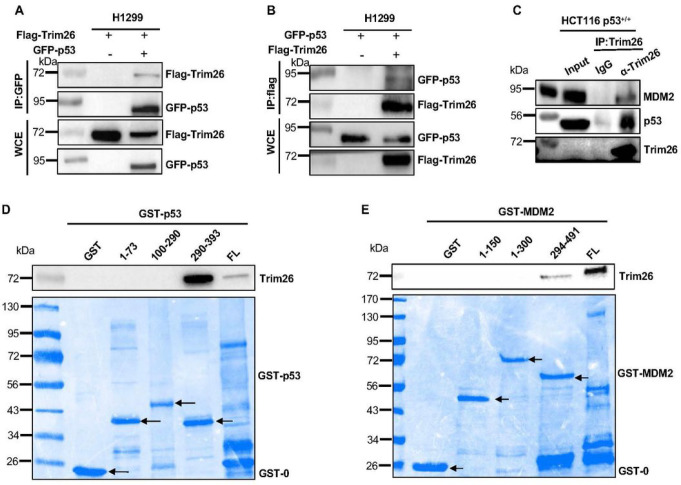
Trim26 interacts with p53 via C-termini. **(A)** H1299 cells were transfected by Flag-Trim26 and with or without GFP-p53 for 48h, using GFP beads to pull down interacted proteins followed by WB analysis with indicated antibodies. **(B)** H1299 cells were transfected by GFP-p53 and with or without Flag-Trim26 for 48h, using Flag beads to pull down interacted proteins followed by WB analysis with indicated antibodies. **(C)** Co-IP-WB analysis shows that exogenous p53 can be pulled down with Trim26 HCT116^(p53+/+)^ cells. Interacted proteins were pulled down by Trim26 antibody followed by WB analysis with indicated antibodies. (**D**) Purified Trim26 is pulled down by GST-p53 fragments. Flag-Trim26 was digested by PreScission Protease and incubated with each of the GST-p53 fragments in beads at RT for 40 min. Upper panel: Bound Trim26 was detected by WB analysis with the Trim26 antibody; lower panel: GST-p53 fragments were stained by Coomassie blue staining. Black arrows indicate GST-p53 fragments bands. (**E**) Purified Trim26 was pulled down by GST-MDM2 fragments. Trim26 was digested by PreScission Protease and incubated with each of the GST-MDM2 fragments in beads at RT for 40 min. Upper: WB analysis of Trim26 as detected by the Trim26 antibody; Lower: GST-MDM2 fragments were stained by Coomassie blue staining. Black arrows indicate GST-MDM2 fragments bands. An equal amount of proteins (50μg) was loaded in each lane.

To better understand if TRIM26 might regulate p53 stability by directly binding to this protein, we performed a set of in vitro and cellular protein-protein interaction assays. First, we conducted reciprocal co-immunoprecipitation (co-IP) assays followed by Western blot analysis after co-overexpressing Flag-TRIM26 with GFP-p53 in p53-deficient H1299 cells (of note H1299 cells were used here because the transfection efficiency is very high). As shown in Fig. 6A, Flag-TRIM26 was co-immunoprecipitated with anti-GFP antibodies in the presence of GFP-p53, but not in the absence of GFP-p53. This result was reproduced reciprocally with anti-Flag antibodies as shown in Fig. 6B. In line with these results, endogenous TRIM26 and p53 were co-immunoprecipitated with anti-TRIM26 antibodies, but not with non-specific IgG (Fig. 6C). Interestingly, MDM2 was also pulled down with the anti-TRIM26 antibodies (Fig. 6C). These results indicate that TRIM26 can interact with both p53 and MDM2.

To map their binding domains, we performed a set of GST-fusion protein-protein interaction assays by using purified GST-p53, GST-MDM2, and their GST-fragments proteins (lower panels of Figs. 6D and 6E). After incubating protein lysates containing TRIM26 with each of these GST-fusion proteins followed by intensive wash with binding buffers as described in the Materials and Methods, the bound TRIM26 was detected by Western blot analysis with anti-TRIM26 antibodies. As shown in the upper panel of Fig. 6D, TRIM26 bound to both the full length p53 and its C-terminal domain (aa291–393) that were fused with GST, but not to GST alone or other GST-p53 fragments. Interestingly, TRIM26 also bound to the full length MDM2 and its C-terminal domain (aa294–494) that were fused with GST, but not other the N- and central domains of MDM2 (Fig. 6E). Intriguingly, TRIM26 appeared to bind to the C-terminus of p53 more strongly than to the full length p53 (last two lanes of upper panel of Fig. 6D) even though the amounts of both the proteins used were equivalent (Fig. 6D, lower panel). This result suggests that the N-terminal and central domains of p53 might have a negative effect on the TRIM26 binding to the C-terminus of p53. This preference was not observed for TRIM26-MDM2 binding (last two lanes of upper panel of Fig. 6E). These results demonstrate that TRIM26 can bind to both p53 and MDM2 via their C-termini directly.

## Discussion

TRIM26 is an under-studied E3 ubiquitin ligase, though it has been shown to play either anti-cancer or oncogenic role in cancer development, pending on cancer types, over the past five years (4, 5). Looking into TRIM26’s role as anti-cancer, multiple studies support this effect. Lu T, Wu et al. conducted research involving overexpressed TRIM26, which led to the downregulation of the pre-apoptosis gene p-AKT in endometrial cancer cells. This intervention resulted in a significant decrease in both tumor volume and weight within the endometrium (21). Similarly, Tao, Luo et al. explored the same pathway of TRIM26’s impact, revealing its ability to suppress tumor growth in the setting of endometrial cancer by downregulating p-AKT expression (5). Another study, conducted by Wang, Chai et al. found that TRIM26, acting as an inhibitor of the PI3K/Akt pathway, effectively suppressed Papillary Thyroid Carcinoma (PTC) (6). Li, Yuan et al. focused on Hepatocellular Carcinoma (HCC) influenced by TRIM26. This study uncovered that the E3 ubiquitin ligase activity of TRIM26 was previously shown to target ZEB1, an oncoprotein crucial for the development of HCC and thus to act as a tumor suppressor in HCC (8). Additionally, another study conducted by Xia, Zheng et al. has confirmed the anticancer behavior of TRIM26 through the downstream regulation of MEK/ERK. This leads to the inhibition of osteosarcoma proliferation (3). Furthermore, in Kidney Clear Cell Carcinoma (KIRC) Shen, Wang et al. discovered that TRIM26 targets SNRBP, which directly participates in ubiquitination. This study reported shorter lifespan in patients with low levels of TRIM26 (22).

Investigating its oncogenic propensity, Xie, Li et al. uncovered that suppressing TRIM26 leads to the inhibition of proliferation, migration, and invasion of bladder cancer cells by impeding the AKT/GSK3b/b-catenin pathway(4). A parallel effect was observed in glioblastoma as revealed by Mahlokozera, Patel et al. who found that TRIM26 stabilizes SOX2 protein and enhances its oncogenic activity in glioblastoma via its C-terminal PRYSPRY domain without engaging its Ring domain and E3 ligase activity (11). Moreover, the oncogenic influence of TRIM26 was also detected in non-small cell lung cancer (NSCLC) by Sun, Lin et al. The study pointed that TRIM26 serves as an ubiquitin ligase for PBX1, and depletion of TRIM26 hindered NSCLC growth (23).

Consistent with the TRIM26 oncogenic role described above, we report here that TRIM26 also plays an oncogenic role in CRC cells and possibly in melanoma cells by inactivating p53. First, overexpression of TRIM26 significantly enhanced the growth and proliferation CRC HCT116 cells (Fig. 1) and of the cells-derived xenograft tumors (Fig. 2). In line with these results, knockdown of TRIM26 reduced the proliferation and colony formation of these cells (Fig. 1). These outcomes appear to be p53-dependent, as the effect of either overexpression or knockdown of TRIM26 on HCT116 cell growth and proliferation was significantly impaired in the absence of p53 (Figs. 1 and 2). Indeed, overexpression of TRIM26 markedly reduced the level and activity of p53 in these cancer cells and the cells-derived xenograft tumor tissues (Figs. 3 and 4). In accordance with these results, knockdown of TRIM26 led to the increase of p53 level and activity as represented in the increase of p21 protein and RNA level. Part of these results were repeated in melanoma cells (SFigs. 2 and 3). Hence, our findings reveal a new role for TRIM26 in negatively regulation p53 stability and activity in CRC cancer cells. By doing so, TRIM26 promotes the proliferation and growth of CRC cells and xenograft tumors derived from human CRC cells.

The E3 ubiquitin ligase activity of TRIM26 targeting ZEB1 and to act as a tumor suppressor in HCC was studied before (8). Surprisingly, we showed here that TRIM26 can ubiquitinate the tumor suppressor p53, consequently leading to the proteolytic degradation of the latter (Figs. 4 and 5). TRIM26 demonstrated its ubiquitin ligase activity targeting p53 by binding to the C-terminal domain of p53 (Fig. 6). This activity was MDM2-independent, as TRIM26 can initiate a basal ubiquitination of p53 in MDM2/p53 double knockout MEF cells (Fig. 5D). But, interestingly, TRIM26 did not appear to compete with MDM2 for binding to and ubiquitinating p53, as p53 ubiquitination was markedly increased in the presence of both TRIM26 and MDM2 (Figs. 5D and 5E). This observation is reasonable, because TRIM26 could bind to the C-terminal domains of both p53 and MDM2 (Figs. 6D and 6E), while MDM2 can bind to the both the C-terminal and the N-terminal domains of p53 with preference to the latter (24). Indeed, a ternary complex containing endogenous MDM2, p53 and TRIM26 was co-immunoprecipitated with anti-TRIM26 antibodies in HCT116 cells (Fig. 6C). These results indicate that TRIM26 can destabilize p53 by binding to and ubiquitinating it either independently or partnering with MDM2.

In summary, our studies as shown here demonstrate that TRIM26 can act as an oncoprotein that promotes the proliferation and growth of CRC cells and xenograft tumor by destabilizing p53 via a ubiquitin-dependent mechanism. Our studies also raise a few outstanding questions. A RING domain-independent function of TRIM26 was recently reported to regulate the stability of SOX via its C-terminal PRYSPRY motif, consequently promoting glioblastoma growth by competing with SOX’s ubiquitin ligase WWP2 (11). Although this study is in line with our findings, supporting the oncogenic role of TRIM26, it also prompted a question of whether TRIM26 might regulate p53 and MDM2 activities via its PRYSPRY motif. Also, is it possible that TRIM26 can promote the growth and proliferation of other types of cancers that harbor wild type p53, such as lung, bladder, melanoma (SFigs. 2 and 3), or breast cancers? Is it possible that TRIM26 might act as a tumor suppressor by degrading mutant p53 in those malignant cancers that harbor mutated p53? Addressing these questions would help us depict a better image for how TRIM26 acts as a dual player in tumorigenesis and as a potential drug target for future development of anti-cancer therapies, at least for gliomas, CRC, bladder cancer and melanoma.

## Conclusion

This study reveals capacity of TRIM26 to promote CRC cell proliferation and xenograft tumor growth, particularly in p53-intact cells displaying oncogenic behavior. By destabilizing p53 through ubiquitination, TRIM26 suppresses p53-dependent apoptosis and interacts closely with both p53 and MDM2. While TRIM26’s oncogenic potential has been recognized in various cancer types, this research contributes novel insights specific to CRC. These findings not only deepen our understanding of TRIM26’s contributions but also presents new possibilities for potential therapeutic strategies, raising interesting questions about its broader impact on different cancer types and its potential as a target for future interventions.

## Materials and Methods

### Plasmids and antibodies

The plasmids encoding Flag-Trim26, PLVX-Flag-Trim26 was generated into the pCDNA3.1 vector, and PLVX vector. The plasmids encoding Flag-p53, HA-MDM2, His-Ub, GST-p53 fragments and GST-MDM2 fragments were described previously (25), (26), (27), (28). Anti-Trim26 (Santa Cruz Biotechnology catalogue no. K3018, 1:1000 dilution), anti-Flag (Sigma-Aldrich, catalogue no. F1804, diluted 1:3,000), anti-p53 (DO-1, Santa Cruz Biotechnology, catalogue no. sc-126, diluted 1:1,000), anti-MDM2(Santa Cruz Biotechnology catalogue no. A2921), anti-p21 (CP74, Neomarkers, Fremont, catalogue no. MS-891-P0, diluted 1:1,000), and anti-GAPDH (Proteintech, catalogue no. 60004-1-Ig), diluted 1:10,000) were commercially purchased. Antibodies against (2A9 and 4B11) were previously described (26), (27).

### Cell culture and transient transfection

Human colorectal cancer, HCT116^(p53+/+)^ and HCT116^(p53−/−)^, lung cancer H1299, p53-null/MDM2-null murine embryonic fibroblast (MEF-DKO) cells, human melanoma SK-Mel5, and SK-Mel-147 cells were used in this study.

In our research endeavors, we were grateful to receive generous contributions from renowned scientists. Dr. Bert Vogelstein from the John Hopkins Medical Institutes kindly provided us with HCT116^p53+/+^ and HCT116^p53−/−^ cells. Additionally, we were fortunate to receive MEF^p53−/−^ and MEF-DKO (p53/MDM2 double knockout) cells as generous gifts from Dr. Guillermina Lozano at MD Anderson Cancer Center, the University of Texas. Human melanoma SK-Mel 5 and SK-Mel 147 cells were obtained from Dr. Shaomeng Wang at University of Michigan. Ensuring the integrity of our research, we conducted STR profiling to confirm the authenticity of the cell lines, while also confirming the absence of mycoplasma contamination.

All the cells were maintained in Dulbecco’s modified Eagle’s medium (DMEM) supplemented with 10% fetal bovine serum, 50 U/ml penicillin and 0.1 mg/ml streptomycin.

All cells were cultured at 37 ° C in a 5% CO 2 humidified atmosphere. Cells seeded on the plate overnight were transfected with plasmids using TurboFect transfection reagent following the manufacturer’s protocol (Thermo Scientific). Cells were harvested at 30–48hour post-transfection for future experiments.

### Generation of stable cell lines

HCT116^(p53+/+)^ and HCT^(p53−/−)^ cells were transfected with PLVX-Flag-Trim26 or the control vector PLVX-Flag-pCDNA. After 48 hours of transfection, the cells were cultured in selection medium containing 500μg/ml Geneticin 418. The selection medium was replaced every 3 days for the next 3 weeks. Subsequently, distinct colonies of surviving cells were transferred onto 6-well plates and the cultures were maintained under the same selection medium, and colonies with overexpression of PLVX-Flag-Trim26were detected by WB analysis using the Flag antibody.

### Cell proliferation assay

IncuCyte S3 Live-Cell Analysis System (Essen Bioscience) was used for kinetic monitoring of cell proliferation. Cells were plated into 96-well plates at a density of 2,000–3,000 cells per well for HCT116^(p53+/+)^ and HCT^(p53−/−)^. Cell proliferation was monitored using IncuCyte S3 live-cell imaging system every 3 h for 5 days. Data were analyzed by the IncuCyte S3 Basic Analyzer software module.

### Colony formation assay

Cells were trypsinized and seeded at equal number of cells on 6-well plates. Media were changed every 3 days until the colonies were visible. Cells were fixed with methanol and stained with crystal violet solution at RT for 30 min. ImageJ was used for quantification of the colonies.

### Mouse xenograft experiments

Ten nude mice were divided randomly into two groups for tumor xenografts as HCT116^(p53+/+)^ and HCT^(p53−/−)^ group. Stable control and Trim26 overexpressed HCT116^(p53+/+)^ and HCT^(p53−/−)^ cells were generated as described above. The 1 × 10^6 control cells or Trim26 overexpressed cells were injected into the flanks of each mouse, at two (left for control and right for overexpression) sides. The tumor size and the mice weights were monitored and recorded every 2 days, and the tumor volume was calculated as mm^3^ = length × (width)^2^ × 0.5 and presented in a graph (Fig. 2C). When the biggest tumor grew up to approximately 1 cm^3^, mice were euthanized. The tumors were harvested, and tumor weights were measured and presented in histograms.

### Immunoprecipitation (IP)

IP was conducted using antibodies as indicated in the figure legends. After the cells were collected and lysed with lysis buffer (50 mM Tris/HCl (pH7.5), 0.5% Nonidet P-40 (NP-40), 1 mM EDTA, 150 mM NaCl, 1 mM dithiothreitol (DTT), 0.2 mM phenylmethylsulfonyl fluoride (PMSF), 10 mM pepstatin A and 1 mM leupeptin). Briefly, 1 mg of proteins were incubated with the indicated antibody at 4°C for 4h or overnight. Protein A or G beads (Santa Cruz Biotechnology) were then added, and the mixture was incubated at 4°C for additional for 2h. Beads were washed at least three times with lysis buffer. Bound proteins were detected by IB with antibodies as indicated in the figure legends.

### Western blotting (WB)

As described previously (29), cells were harvested and lysed in lysis buffer consisting of 50 mM Tris/HCl (pH7.5), 0.5% Nonidet P-40 (NP-40), 1 mM EDTA, 150 mM NaCl, 1 mM dithiothreitol (DTT), 0.2 mM phenylmethylsulfonyl fluoride (PMSF), 10 mM pepstatin A and 1 mM leupeptin. Equal amounts of clear cell lysate (30–50mg) were used for WB analyses.

### GST fusion protein association assay

GST-tagged MDM2 fragments or p53 fragments were expressed in E. coli and conjugated with glutathione-Sepharose 4B beads (Sigma-Aldrich). Protein-protein interaction assays were conducted by using cell lysates with Flag-Trim26. Briefly, the cell lysates with Flag-Trim26 were incubated and gently rotated with the glutathione-Sepharose 4B beads containing 500 ng of GST-MDM2 fragments, GST-p53 fragments or GST only at RT for 40 min. The mixtures were washed three times with GST lysis buffer (50 mM Tris/HCl pH 8.0, 0.5% NP-40, 1 mM EDTA, 150 mM NaCl, 10% glycerol). Bound proteins were analyzed by WB with the antibodies as indicated in the figure legends.

### In vivo ubiquitination assay

H1299, or p53^−/−^/MDM2^−/−^ MEF cells were transfected with plasmids encoding Flag-p53, HA-MDM2, His-Ub or Falg-Trim26 as indicated in the figure legends. At 42 hr after transfection, cells were treated with 20μM MG132 for 6 h and then harvested and split into two aliquots, one for WB analysis and the other for ubiquitination assay. Briefly, cell pellets were lysed in buffer I (8 M urea, 0.1 M Na_2_HPO_4_/NaH_2_PO_4_ (pH 8.0), 10 mM Tris-HCl (pH 8.0), 200 mM imidazole, 10 mM β-mercaptoethanol) and incubated with Ni-NTA beads (Qiagen) at room temperature for 4 hr. Beads were washed twice with buffer I and buffer II (8 M urea, 0.1 M Na_2_HPO_4_/NaH_2_PO_4_ (pH 6.3), 10 mM Tris-HCl (pH 6.3), 10 mM β-mercaptoethanol). Proteins were eluted from beads in buffer III (200 mM imidazole, 0.15 M Tris-HCl (pH 6.7), 30% glycerol, 0.72 M β-mercaptoethanol, and 5% SDS). Eluted proteins were detected by WB analysis with indicated antibodies. H1299 cells and p53^−/−^/MDM2^−/−^ MEF cells were used to determine the ubiquitination of endogenous p53 in the presence or absence of Trim26 or MDM2 using the same assay as described above and also briefed in the legends for Fig. 6.

### Reverse transcription and quantitative PCR analyses

Total RNA was isolated from cells using Trizol (Invitrogen, Carlsbad, CA, USA) following the manufacturer’s protocol. Total RNAs of 0.5 to 1 mg were used as templates for reverse transcription using poly-(T) 20 primers and M-MLV reverse transcriptase (Promega, Madison, WI, USA). Quantitative PCR (qPCR) was conducted using SYBR Green Mix according to the manufacturer’s protocol (BioRad, Hercules, CA, USA). The primers for human Trim26 and p21 and P53 are as follows: Trim26, F:5’-GAACCACCTGAGTACCCTAAGG-3’;R: 5’-CTCAGCCACAATGTACTGCCTC-3’. The primers for human p53, p21, were used as previously described(30, 31).

### Statistics

All in vitro experiments were performed in biological triplicate. The student’s two-tailed t-test was used to determine mean difference among groups. P < 0.05 was considered statistically significant. Data are presented as mean ± s.e.m.
